# 

**Published:** 1896-09

**Authors:** 


SEPTEMBER, 1896.

*Vol. XIV.* *No.* **53.**

THE

### BRISTOL MEDICO-CHIRURGICAL JOURNAL

**A Quarterly Journal of the Medical Sciences for the West of England and South Wales**

*PUBLISHED UNDER THE AUSPICES OF*

*THE BRISTOL MEDICO-CHIRURGICAL SOCIETY.*

"Scire est nescire, nisi id me

Scire alius sciret."

LIBRARY OF CONGRESS
37477
DEC 1 1897
SMITHSONIAN DEPOSIT.

*Editor:* R. SHINGLETON SMITH, M.D., B.Sc.

WITH WHOM ARE ASSOCIATED

J. MICHELL CLARKE, M.A., M.D.

F. R. CROSS, M.B. AND W. H. HARSANT.

*Assistant-Editor:* L. M. GRIFFITHS.

*Editorial Secretary:* B. M. H. ROGERS, B.A., M.D., B.CH.

BRISTOL: J. W. ARROWSMITH;

LONDON: J. & A. CHURCHILL, 7 GREAT MARLBOROUGH STREET.

*Price One Shilling and Sixpence.*

